# Molecular evolution of measles viruses circulated in Taiwan 1992-2008

**DOI:** 10.1186/1743-422X-6-219

**Published:** 2009-12-10

**Authors:** Wen-Yueh Cheng, Lili Lee, Paul A Rota, Dustin Chen-Fu Yang

**Affiliations:** 1 Research and Diagnostic Center, Centers for Disease Control, DOH, Taiwan; 2 Measles, Mumps, Rubella, and Herpesviruses Laboratory Branch, Centers for Disease Control and Prevention, Atlanta, GA, USA

## Abstract

Genetic analyses of viral samples from 74 laboratory confirmed measles cases occurring in Taiwan during 1992-2008 identified six viral genotypes D3, D5, D9, G2, H1 and H2. The most frequently detected genotype, H1, was associated with outbreaks in 1994 and 2002, and was the likely indigenous genotype in 1992. In response to the outbreaks, two catch-up campaigns were launched and a routine second dose of measles, mumps, and rubella vaccine at entry to elementary school was introduced. The vaccination campaigns successfully reduced the number of measles cases in Taiwan, and many of the more recent cases can be traced to importations, primarily from other Asian countries. A number of measles genotypes which were associated with outbreaks in other Asian countries were detected among the more recent cases. The more recent genotype H1 viruses had sequences that were identical to those currently circulating in China or associated with international importation of virus.

## Background

Measles is a contagious human disease caused by measles virus (MeV). Symptoms include high fever, conjunctivitis, coryza, cough, the appearance of Koplik spots on the buccal mucosa and a maculopapular rash. Vaccination programs have dramatically reduced the incidence of measles on a global scale [1,2]. Measles transmission has been interrupted in the region of the Americas, and the Eastern Mediterranean, European and Western Pacific Regions have established elimination targets for the near future. Despite the success of global measles vaccination programs, measles is still responsible for an estimated 245,000 deaths each year [3], with most of these deaths occurring in developing countries. Sustained measles outbreaks continue to occur in developed countries that have failed to maintain a high level of population immunity [4].

As part of laboratory-based surveillance for measles, genetic characterization of circulating wild-type viruses provides an important tool for mapping transmission routes, documenting the elimination of endemic strains, and distinguishing vaccine reactions from wild-type infections [5-11]. In a given country or region, a genotype is considered endemic if it is consistently associated with outbreaks over an extended time period. On the contrary, the identification of multiple genotypes associated with a limited number of outbreaks and/or sporadic cases is more consistent with multiple importations of virus than with endemic virus circulation. This pattern of measles circulation was described for the United States, Australia, and Canada during the last decade [7,8,10,12,13].

Live attenuated measles vaccine was first introduced to Taiwan in 1968, but no mass vaccination policy was established. From 1978 to 1987, the vaccine was provided to infants at both 9 and 15 months of age. In early 1988, vaccination was scheduled for 12-months of age, but a two-dose schedule (9 months and 15 months) was initiated later in the year because an outbreak occurred, and this policy continued until 1991. In 1992, one dose of measles vaccine was given at 9-months and a second dose of measles, mumps, and rubella vaccine (MMR) was administered at 15-months. Two catch-up campaigns were implemented to improve coverage rates in response to small outbreaks. From 1992 to 1994, the campaign targeted junior high school through preschool children (birth cohort 1976/09 to 1990/09), while the campaign of 2001 to 2004 was aimed at elementary school students (birth cohort 1990/09 to 1994/09). The current two-dose MMR program for 12-15 month old infants and first graders (6 years old) was implemented in 2006 [14]. The coverage rates with the first dose of measles vaccine among children aged 13-24 months was 84% in 1993-94, and the second dose MMR coverage rate improved from 69% in 1993-94 to 80-85% in 1995. Verifying immunization for all entrants to primary school started in 1991, and by 1995, the overall vaccine coverage with at least one dose of MMR in primary schoolchildren was 96% [15].

In 1985, measles was listed as a reportable disease in Taiwan, but routine serologic confirmation of infection was not started until 1991. In 2000, to strengthen measles surveillance, laboratory confirmation was required for all suspected cases. Starting in 2002, clinical specimens including throat swabs, urine, and whole blood were also obtained for virological surveillance.

In this study, the viral genotypes associated with 74 confirmed cases of measles that occurred in Taiwan between 1992 and 2008 were determined.

Sixteen sequences were obtained from viral isolates and the others were obtained directly from clinical samples. Therefore, this is the first report that describes the molecular epidemiology of MeV in Taiwan.

## Results

### Epidemiology

**Figure 1:**
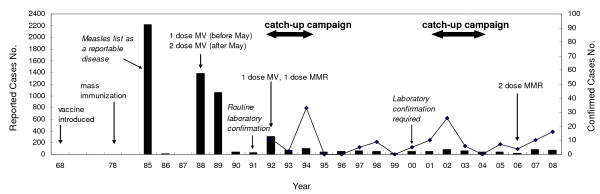
**Measles surveillance and vaccination in Taiwan, 1968-2008**. Figure shows numbers of reported cases (black bars) and laboratory confirmed cases (lines) by year. The dates of introduction of measles vaccination and changes in the measles vaccination program, including campaigns, are indicated by the text and arrows. The year when measles became a reportable disease was 1985. The year when routine laboratory confirmation started was 1991, and the year when laboratory confirmation required was 2000.

Measles was listed as a reportable disease since 1985 and information regarding measles infections before 1985 is incomplete. From 1985 to 1992, four major measles outbreaks occurred in Taiwan (Figure 1) in 1985 (2,219 cases), 1988 (1,386 cases), 1989 (1,060 cases) and 1992 (303 cases). The epidemic in 1985 resulted in 97 deaths with reported case fatality rate of 4.4%, whereas the epidemic of 1988-89 was associated with 12 deaths and a case fatality rate of 0.5%. The high mortality rates associated with the outbreaks in 1985 and 1989 were likely due to under reporting of the cases. In an outbreak investigation among elementary and junior high schools in northeastern Taiwan in 1989, the reporting of measles by physicians was only 6.1% [16]. Beginning in 1993, the annual number of reported measles cases was below 100, the annual number of laboratory confirmed measles cases averaged 8.6, with a range of 0-33 cases. There were no laboratory confirmed cases for years of 1995, 1996, 1999, and 2004 (Figure 1).

### Genotyping

Genetic analysis was conducted on 74 measles cases (Table 1) and 4 vaccine reactions that occurred between 1992 and 2008. Genotype information on MeVs that circulated in Taiwan before 1992 is not available. The six genotypes were detected among the 74 measles cases included D3, D5, D9, G2, H1, and H2 (Table 1). Four sequences from genotype A were obtained from patients who presented with rash following vaccination.

**Table 1:** Genetic characterization of measles viruses in Taiwan, 1992-2008

Strain Designation		Age (yy/mm)	Specimen	Genotype	Accession number^a^	Vaccination	Comment
MVs/Taiwan/18.92/1		00/10	serum	H1	AY737397	Unknown	

MVs/Taiwan/18.92/2		10/02	serum	D5	AY737398	Unknown	

MVs/Keelung.TWN/18.92/3		04/02	serum	H1	EU914228	Unknown	

MVs/Taiwan/19.92		13/06	serum	D3	EU914241	Unknown	

MVs/Taiwan/22.92/1		01/09	serum	H1	AY737399	Unknown	

MVs/Taiwan/22.92/2		unknown	serum	H1	AY737400	Unknown	

MVs/Taipei.TWN/11.93		02/10	serum	H1	DQ380233	Unknown	

MVs/Keelung.TWN/23.93		01/09	serum	H1	EU914229	Unknown	

MVi/Taoyuan.TWN/21.94		03/10	throat swab	H1	AY737401	Unknown	1994 Taoyuan measles outbreak

MVi/Taoyuan.TWN/22.94		05/10	whole blood	H1	AY737402	Unknown	1994 Taoyuan measles outbreak

MVi/Taoyuan.TWN/24.94/1		02/10	throat swab	H1	AY737403	Unknown	1994 Taoyuan measles outbreak

MVi/Taoyuan.TWN/24.94/2		03/08	whole blood	H2	AY737404	Unknown	1994 Taoyuan measles outbreak

MVi/Taoyuan.TWN/24.94/3		03/10	throat swab	H1	AY737405	Unknown	1994 Taoyuan measles outbreak

MVs/Taichung.TWN/21.97/1		01/00	serum	G2	AY737406	Unknown	

MVs/Taichung.TWN/21.97/2		00/05	serum	G2	AY737407	No	

MVs/Taichung.TWN/23.97		10/08	serum	G2	AY737408	Unknown	

MVs/Taipei.TWN/36.97		01/02	serum	H1	AY737409	Unknown	

MVs/Chiayi.TWN/13.98		01/07	serum	H2	EU914239	Unknown	Nosocomial

MVs/Chiayi.TWN/16.98		00/10	serum	H2	EU914240	Yes	Nosocomial

MVs/Taipei.TWN/11.00		00/10	serum	D3	EU914242	No	

MVs/Hualian.TWN/12.00		00/11	serum	D5	AY737410	No	

MVs/Taitung.TWN/21.00		27/07	serum	H1	EU914230	Unknown	

MVs/Taipei.TWN/07.01		28/09	serum	H1	AY737411	Unknown	Imported from China

MVs/Hsinchu.TWN/11.01/1		00/09	serum	H1	AY737412	No	

MVs/Hsinchu.TWN/11.01/2		00/08	serum	H1	AY737413	No	

MVs/Taipei.TWN/16.01		22/00	serum	H1	AY737414	Unknown	

MVs/Taoyuan.TWN/29.01		00/10	serum	H1	AY737415	No	Imported from China

MVs/Taoyuan.TWN/45.01		00/09	serum	H1	AY737416	No	Imported from China

MVs/Taichung.TWN/10.02		01/00	serum	D3	AY737417	No	Imported from Philippines

MVs/Taipei.TWN/14.02		09/10	serum	H1	AY737418	Yes	

MVs/Kaohsiung.TWN/16.02		29/04	serum	D5	AY737419	No	

MVs/Taipei.TWN/26.02		00/07	urine	H1	AY737420	No	Imported from China

MVs/Taipei.TWN/27.02		00/10	throat swab	H1	AY737421	No	Imported from China

MVs/Pingtung.TWN/33.02		00/06	urine	H1	AY737422	No	Imported from China

MVs/Miaoli.TWN/34.02		27/04	urine	H1	EU914225	Yes	

MVs/Taichung.TWN/36.02/1		00/07	throat swab	H1	AY737423	No	

MVs/Taipei.TWN/36.02/2		26/01	urine	H1	EU914227	Yes	

MVs/Taichung.TWN/38.02		14/10	urine	H1	AY737424	Yes	Taichung outbreak, school clustering

MVs/Taichung.TWN/39.02/1		09/11	urine	H1	EU914226	No	Taichung outbreak, school clustering

MVs/Hsinchu.TWN/39.02/2		00/01	serum	H1	AY737425	No	

MVi/Taichung.TWN/40.02/1		13/02	urine	H1	AY737427	Yes	Taichung outbreak, school clustering

MVi/Taichung.TWN/40.02/2		09/11	urine	H1	AY737428	Yes	Taichung outbreak, school clustering

MVi/Taichung.TWN/40.02/3		13/03	whole blood	H1	AY737429	Yes	Taichung outbreak, school clustering

MVs/Hsinchu.TWN/40.02/4		29/00	serum	H1	AY737426	Unknown	

MVs/Taichung.TWN/41.02		13/11	urine	H1	EU914244	Yes	Taichung outbreak, school clustering

MVs/Hualian.TWN/18.03/1		00/10	serum	D3	EU914243	No	Philippines native

MVi/Hualian.TWN/18.03/2		00/10	whole blood	D3	AY738084	No	Philippines native

MVi/Taoyuan.TWN/20.03		00/11	throat swab	H1	AY738085	No	Imported from China

MVs/Taichung.TWN/45.03		28/10	urine	D5	AY738086	Yes	Imported from Thailand

MVs/Yilan.TWN/48.03		25/01	throat swab/urine	D9	AY738087	Unknown	

MVi/Taipei.TWN/09.05		01/02	throat swab	H1	EU914232	No	Household

MVs/Taipei.TWN/10.05		02/00	urine	H1	EU914231	No	Household

MVs/Taipei.TWN/46.05		40/04	urine	D5	EU914244	No	German native

MVs/Taipei.TWN/17.06		00/10	throat swab/urine	H1	EU914233	No	Imported from China

MVs/Tainan.TWN/21.06		00/10	urine	H1	EU914234	No	Imported from China

MVs/Taipei.TWN/39.06		25/08	throat swab/urine	H1	EU914235	No	Imported from China

MVs/Tainan.TWN/23/07/1		30/09	throat swab/urine	D5	EU914245	No	Imported from Japan

MVi/Taipei.TWN/23.07/2		26/05	throat swab/urine	D5	EU914246	Unknown	Imported from Japan

MVi/Taipei.TWN/30.07		00/10	urine	H1	EU914236	No	Imported from China

MVs/Tainan.TWN/31.07/1		00/07	urine	H1	EU914237	No	Imported from China

MVs/Tainan.TWN/33.07		01/02	throat swab/urine	H1	EU914238	No	Imported from China

MVs/Taipei.TWN/34.07		16/05	throat swab/urine	D5	EU914247	Unknown	Imported from Japan

MVs/Hualian.TWN/01.08		04/11	throat swab	D9	GQ338669	No	Imported from Philippines

MVi/Taipei.TWN/05.08		27/00	urine	H1	GQ338670	Unknown	Imported from China

MVs/Chayi.TWN/11.08		00/10	throat swab/urine	H1	GQ338671	No	Imported from China

MVs/Taipei.TWN/16.08		45/07	throat swab/urine	H1	GQ338672	Unknown	Imported from China

MVi/Taipei.TWN/17.08		32/07	urine	D5	GQ338673	Unknown	Imported from Japan

MVs/Taoyuan.TWN/29.08		22/11	throat swab	H1	GQ338674	Unknown	

MVs/Taipei.TWN/34.08/1		25/04	urine	D9	GQ338675	Unknown	Imported from Thailand

MVs/Taipei.TWN/34.08/2		00/10	throat swab/urine	D9	GQ338676	No	Household

MVs/Kaohsiung.TWN/45.08		01/05	throat swab	H1	GQ338677	No	Imported from China

MVs/Kaohsiung.TWN/47.08		04/05	throat swab	H1	GQ338678	No	Nosocomial

MVs/Kaohsiung.TWN/52.08		00/09	throat swab/urine	H1	GQ338679	No	Nosocomial

MVi/Kaohsiung.TWN/53.08		39/11	urine	H1	GQ338680	No	Nosocomial

^a^ Indicates N gene Genbank accession number

### Molecular characterization

**Figure 2:**
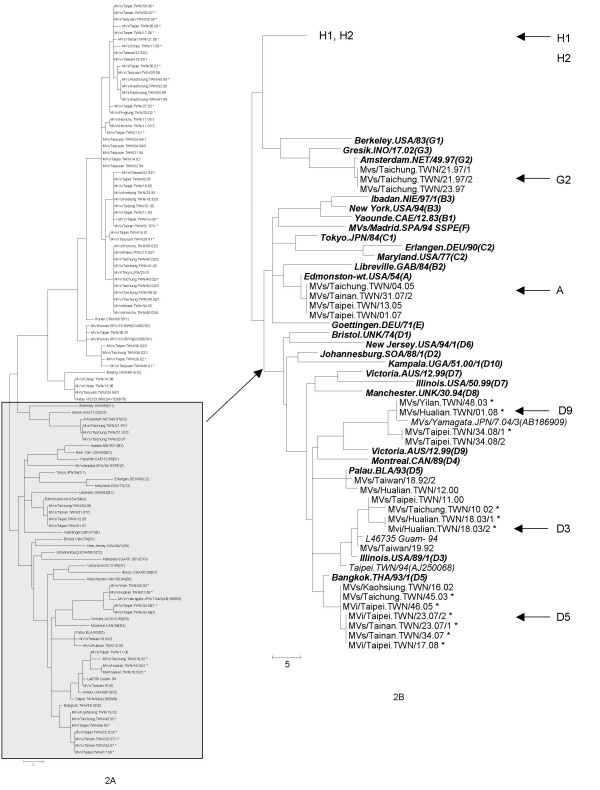
**Phylogenetic analysis of the carboxyl terminal 456 nucleotides of the N gene sequences from measles virus cases in Taiwan, 1992-2008**. The dendrogram was created with MEGA 4.1 by the neighbor-joining method with 1000 bootstrap replicates. The asterisks indicate cases imported from foreign countries. Several sequences with highest nucleotide blast search from GenBank (shown by italic) are also included. The reference strains recommended by WHO (shown by bold italic) also included for comparison. The tree on 2A shows all sequences with the part of the tree showing all genotypes except genotypes H1 and H2 expanded for easier viewing on 2B. Genotypes detected in Taiwan are indicated on the tree of 2B.

Genotype D3 was detected in five cases, and three of these (1 in 2002, 2 in 2003) were travelers from the Philippines (Figure 2). All of the genotype D3 sequences were placed within the same cluster as the reference strain, Illinois. USA/89.1, and more divergent from an earlier D3 sequence, MVs/Taipei.TWN/94 (AJ250068) [17], which was closely related to the Japanese strains (D87485, D87486, and D87490) detected in 1989.

Genotype D5 was detected in nine cases in Taiwan. Genotype D5 has been reported to be circulating in Thailand and had been associated with cases in Japan and imported cases in the USA [18,19]. The source of virus for three of the cases (one each in 1992, 2000, and 2002) was not identified. The other six cases had sequences that were more closely related to the Bangkok reference strain than to the Palau reference strain (Figure 2). Of these six cases with genotype D5 detected, one case was imported from Thailand, one from Germany, and the four more recent cases in 2007-2008 were imported from Japan.

The genotype D9 sequence from 2003 was obtained from a 25-year-old woman who had traveled to Japan for 10 days before onset of clinicalsymptoms. Three other D9 sequences were obtained in 2008, one was from 4 year old girl who traveled to the Philippines 13 days before rash appeared. The other two cases from 2008 were from the same household including a 25 year old man who had traveled to Thailand 10 days before rash onset and then transmitted virus to his 10 months old niece. Genotype D9 was initially detected in East Timor and Java, Indonesia [20,21] and has recently been associated with outbreaks in Japan [22].

Genotype G2 was first identified in 1997 in Indonesia and Malaysia [23,24]. The three genotype G2 viruses detected in Taiwan in 1997 shared identical sequences and were closely related to the sequence of the reference strain for genotype G2, MVi/Amsterdam.NET/49.97 (AF171232), which was isolated from secondary cases of measles associated with an index case from Indonesia. Though the source of the virus was not identified, a linkage to Indonesia or Malaysia is speculated (Figure 2).

**Figure 3:**
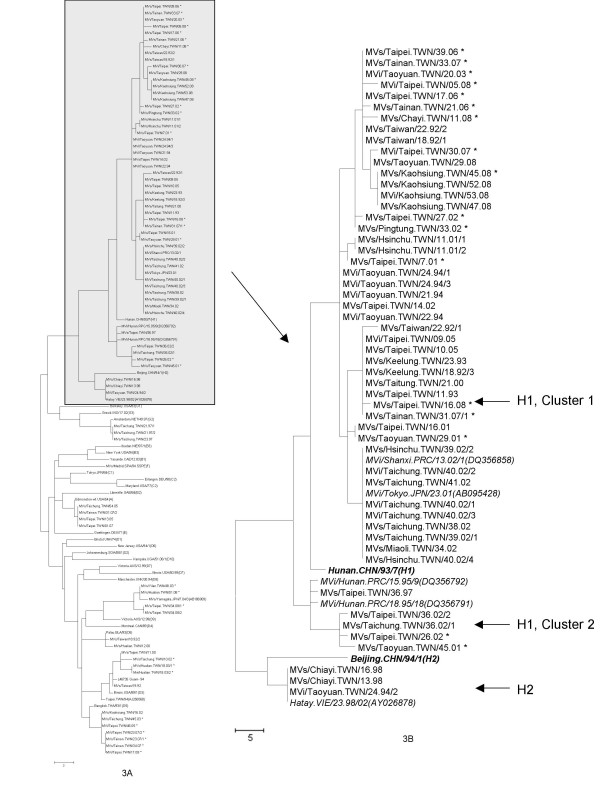
**Phylogenetic analysis of the 456 nts coding for the COOH terminus of the N protein of measles viruses detected in Taiwan, 1992-2008, highlighting the sequences in genotype H1**. The dendrogram was created with MEGA 4.1 by the neighbor-joining method with 1000 bootstrap replicates. The asterisks indicate viruses detected from imported cases. Several closely related sequences with the highest nucleotide BLAST scores from GenBank are also included (italic). The reference strains recommended by WHO also included for comparison (bold italic). The tree in figure 3A shows all sequences with the part of the tree showing only genotypes H1 and H2 expanded for easier viewing in figure 3B. The two previously described clusters of genotype H1 [25], are represented by MVi/Shanxi.PRC/13.02/1 (DQ356858) for cluster 1 and MVi/Hunan.PRC/18.95/18 (DQ356791) and MVi/Hunan.PRC/15.95/9 (DQ356792) for cluster 2.

Genotype H1 was detected in 50 of the 74 samples from cases occurring during 1992-2008. In 1992, genotype H1 sequences belonged to two lineages within what has been described as genotype H1, cluster 1 [25] (Figure 3). Two H1 sequences obtained in 1993 were identical to one of the sequences detected during the previous year (MVs/Keelung.TWN/18.92/3). Four identical genotype H1 sequences were obtained from the Taoyuan area in 1994. The only H1 sequence detected in 1997, MVs/Taipei.TWN/36.97, was very closely related (99% identity) to Chinese viruses detected in 1995, MVi/Hunan.PRC/15.95/9 (DQ356792) and MVi/Hunan.PRC/18.95/18 (DQ356791). One genotype H1 sequence was detected in 2000, but information regarding the source of infection was not available. During 2001, six cases were confirmed as genotype H1. Three of these cases had traveled to China 7-21 days before onset of rash and the sequences from the six cases were distributed over three different lineages (Figure 3).

There were 26 measles cases confirmed by serology in 2002, and 10 cases were from a school outbreak that occurred from September to October. There were 17 cases with sequence information and 15 of the H1 sequences belonged to 3 different lineages. Of those, 3 were imported from China (MVs/Taipei.TWN/26.02, MVs/Taipei.TWN/27.02, and MVs/Pingtung.TWN/33.02). Nine of the H1 isolates associated with the 2002 outbreak (six of these were either household or school clustering) had identical sequences and formed a separate lineage (Table 1, Figure 3) which included identical sequences from viruses isolated from Japan and China, MVi/Tokyo.JPN/23.01 (AB095428), MVi/Fuji.JPN/21.02 (AB095430), MVi/Kawasaki.JPN/36.01 (AB095429), MVi/Shanxi.PRC/13.02/1 (DQ356858), and MVi/Shanghai.PRC/30.02/1 (DQ356839), suggesting that these viruses were introduced by importation.

The genotype H1 sequence detected in 2003 was from an eleven months old infant returning from a visit to China. The two cases with genotype H1 in 2005 were in the same family and there was no history of travel. The six genotype H1 sequences detected in 2006-2007 were all imported from China (Table 1). Among the eight genotype H1 sequences detected in 2008 (Table 1), four had a travel history to China. The case associated with MVs/Kaohsiung.TWN/45.08 was responsible for nosocomial transmission in two hospitals and the index case represented by MVs/Kaohsiung/52.08 was identified later. In this outbreak, eight cases were confirmed and only four had adequate samples for PCR testing. One of the genotype H1 sequences obtained in 2008, MVs/Taoyuan.TWN/29.08, was detected by the laboratory surveillance system but no source was identified. A search of GenBank found an identical sequence from case reported in Hong Kong, MVs/HongKong.CHN/17.08 (EU870594). Therefore, there were multiple introductions of genotype H1 viruses into Taiwan from China and the epidemiologic information documented importation for 17 of 50 cases. The sequences from the recent genotype H1 viruses that were imported into Taiwan showed 100% sequence identity with genotype H1 viruses that were circulating in China or were associated with international importation of virus. For example, MVs/Tainan.TWN/33.07 (EU914238) had 100% identity with MVi/Sichuan.PRC/28.04/1 (EU557230) and MVi/Chongqing.PRC/20.04/1 (EU557209). MVs/Tainan.TWN/31.07/1 (EU914237) shared identical sequences with MVs/Aberdeen.GRB/10.06 (EF079131), MVi/Shanghai.PRC/22.06/11 (DQ902857) and MVs/Oregon.USA/8.06 (DQ888762).

Among the 50 H1 sequences characterized, forty-five cases were clustered with the reference strain for genotype H1 (Figure 3) and the other five cases, displayed in a separate lineage, were closely related to two genotype H1 viruses from China, MVi/Hunan.PRC/18.95/18 (DQ356791) and MVi/Hunan.PRC/15.95/9 (DQ356792).

Only three cases were confirmed to be associated with genotype H2, one was in 1994 from the Taoyuan outbreak and the other two were from an outbreak in a hospital in 1998. Although no information regarding the source of these cases was available, these are more closely related to the isolates from Vietnam [26].

## Discussion

Historically, clade H of MeV was associated with Asian countries, with genotype H1 being prevalent in China [27], and genotype H2 linked to Vietnam [26]. Baseline virological surveillance was not conducted in many Asian countries so the exact distribution of the clade H viruses in the pre-vaccine era is unknown.

This report contains the first analysis of the genetic characteristics of MeVs circulating in Taiwan over the seventeen year period from 1992 to 2008. In Taiwan, measles vaccination started in 1978, but epidemiologic information was not available until 1991. Laboratory-based surveillance was initiated in 1991, but routine virological sampling did not occur until 2002. Before 2000, only serum samples were collected and the percentage of sampling from all reported cases was variable (range: 52% to 85%). The low sampling rate from 1995, 1996, and 1999, of 59.5%, 59.6%, and 52.2% respectively, could have contributed to the absence of confirmed measles cases for those years.

Even though virological surveillance was not conducted in Taiwan before 1992, the data presented here suggest that MeV genotype H1, the most frequently detected genotype in the study, represented the indigenous genotype in Taiwan. Genotype H1 viruses were detected almost every year beginning in 1992 with the exception of 1995, 1996, 1998, 1999, and 2004 (Table 1). Importation of measles from China was evident beginning in 2001 and accounted for 10 of 14 detections of genotype H1 after 2006. It is interesting to note that genotype H1 viruses were detected in Taiwan before they were documented in China. It is also possible that genotype D3, which was an indigenous genotype in Asian countries including the Philippines, could have been an endemic genotype in Taiwan at one time [17] although D3 was only detected twice in 1992 and 2000.

Recent virological surveillance has documented the spread of MeV in the Western Pacific Region. A large measles outbreak that occurred in Korea in 2000 was caused by genotype H1 viruses [28] and genotypes H1 and D9 were imported into Japan [22,29-32]. The most recent outbreaks in Japan were due to importation of genotype D5 [33]. This report documents the importation of MeV into Taiwan which began to introduce the foreign laborers from countries of Indonesia, Malaysia, Philippines, Thailand, and Vietnam in the beginning of 1989. The number of foreign workers reached 330,000 by end of 2006, accounting for 1.4% of the total population. The multiple genotypes and sporadic cases detected after 2003, except for two cases in 2005, and one in 2008, were all documented to be the result of importation and the genotypes detected, D3, D5, D9, G2, H1 and H2, are or were circulating in nearby Asian countries. The observation that two genotypes, H1 (4 cases) and H2 (1 case), were detected in the 1994 outbreak implied that there were at least two different chains of transmission associated with the outbreak.

In the dendrogram derived from the N gene sequences (Figure 3), two clusters of sequences within genotype H1 were observed. The majority of the sequences were more closely related to the H1 reference strain and related viruses have been detected on an ongoing basis in China where this group of viruses is referred to as cluster 1 [25]. Five of the sequences from Taiwan, including MVs/Taipei.TWN/36.97, MVs/Taoyuan.TWN/45.01, MVs/Taipei.TWN/26.02, MVs/Taichung.TWN/36.02/1, and MVs/Taipei.TWN/36.02/2, were clustered together and were more distinct from H1 reference strain. This group of viruses has also been detected in China and is referred to as cluster 2 [25,34].

The diversity of genotypes and the low number of cases in Taiwan is similar to what has been described in other countries that are in elimination phases of measles control [9,10,12,13]. The situation in Taiwan is different from some other countries in the elimination phase because the genotype H1 is continually being reintroduced by importation from neighboring Asian countries, so it is difficult to document interruption of transmission of the suspected indigenous genotype. However, both molecular and epidemiologic data were used to link the more recently detected genotype H1 viruses to international importation. While virological surveillance will help to document the sources of measles cases, formal documentation of elimination will depend on meeting a number of criteria, from both laboratory-based and epidemiologic studies. Of course, the nosocomial measles outbreak that occurred in 2008 highlighted the vulnerability of infants younger than 12 months of age, unimmunized adults, and the high risk of importation associated with international travel. These events underscored the importance of strengthening immunization programs, disease surveillance, and laboratory confirmation.

## Conclusion

This study reported the first analysis of the genetic characteristics of MeVs circulating in Taiwan over the seventeen year period from 1992 to 2008. Genetic analyses of viral samples from laboratory confirmed measles cases identified six viral genotypes D3, D5, D9, G2, H1 and H2. Genotype H1 remains the most frequently encountered MeV transmitted among Asian countries. Recent measles cases were epidemiologically linked to the importation from foreign countries. These results highlight the importance of integration of immunization programs, disease surveillance systems, and laboratory diagnosis.

## Materials and methods

### Clinical Specimens

Specimens including throat swabs, urine, whole blood, or serum were collected from suspected cases and tested in the laboratory at the Taiwan CDC. During the period of 1992-2008, various specimens from 896 suspected patients were collected for laboratory tests. Specimens were processed with RT-PCR amplification from a total of 174 patients, including 42 with only one serum collected at the acute phase and tested negative for measles IgM and IgG, the other 132 cases were serologically confirmed with positive measles IgM. Finally, 74 confirmed measles cases, including 16 virus isolates, were sequenced and used for genetic characterization. Here, a confirmed case is defined serologically either by a positive IgM, or a significant rise (4× or higher) of IgG titer between acute and convalescence phase, or a positive RT-PCR result from various clinical samples (serum, throat swab or urine). Any confirmed case that has travel history abroad 7-23 days before onset of rash is classified as an imported case [35].

### Serological Testing

Serum specimens were tested for measles IgM and IgG antibodies using Enzygnost Anti-Marsen-Virus/IgM, Enzygnost Anti-Marsen-Virus/IgG (Dade Behring, Marburg, Germany) following the manufacturer's instructions.

### Virus Culture

Clinical specimens including throat swab, urine sediments and lymphocytes were inoculated onto B95a cells, a marmoset B lymphoblastoid cell line transformed by Epstein-Barr virus [36], and observed for the presence of cytopathic effect (CPE). Inoculated cells were blind passaged up to two times before discarding those with no evident CPE.

### Molecular Analysis of Measles Virus

RNA was extracted from infected cells or directly from clinical specimens by using the Viral RNA mini kit (Qiagen Inc., Chatsworth, CA) following the manufacturer's instructions. To obtain the sequence of the 450 nt region of the measles N gene that is required for genotyping, RT-PCR was performed by using a one-step RT-PCR kit (Qiagen) with reverse primer MV64 (nt 1719-1739, 5'-TATAACAATGATGGAGGGTAG-3') and forward primer MV59 (nt 866-889., 5'-GATATGTGACATTGATACATATAT-3'). Primer concentration was 0.4 μM each. The PCR cycling conditions were reverse transcription at 50°C for 30 min, initial PCR activation step by 95°C for 15 min, followed by 35 cycles of 30 s at 94°C, 30 s at 51°C and 1 min at 72°C, with a final extension for 5 min at 72°C. A nested PCR was then performed on the resulting PCR product of 873 bps using HotStartTaq Master Mix Kit (Qiagen) with primers MV60 and MV63 (0.2 μM each) as described elsewhere [37], and the cycling conditions were 95°C for 15 min followed by 30 cycles of 30 s at 94°C, 30 s at 63°C and 1 min at 72°C, with a final extension for 5 min at 72°C. Sequencing reactions were performed by using the BigDye Terminator Cycle Sequencing Ready Reaction kit (Applied Biosystems, Forster City, CA) with the same primers as those used for the nested PCR. Nucleotide sequences were analyzed with MegAlign version 3.1.7. Phylogenetic trees were drawn with MEGA version 4.1 by neighbor-joining using 1000 bootstrap replicates. The wild-type MeV isolates and genotype sequences from Taiwan were named as recommended by WHO [38]. The WHO-designated reference sequences [39] for each genotype were obtained from GenBank.

## Abbreviations

MeV: measles virus; MMR: measles, mumps, and rubella; RT-PCR: reverse transcription-polymerase chain reaction; CPE: cytopathic effect; N: Nucleoprotein.

## Competing interests

The authors declare that they have no competing interests.

## Authors' contributions

WYC carried out most of the studies and drafted the manuscript. LL participated parts of the studies and writing. PAR provided consultation and editing of the manuscript. DCY provided consultation and preparation of the final report. All authors read and approved the final manuscript.
